# Supplementary material for: Effectiveness of Exercise, Cognitive Behavioral Therapy, and Pharmacotherapy on Improving Sleep in Adults with Chronic Insomnia: A Systematic Review and Network Meta-Analysis of Randomized Controlled Trials
Source: Healthcare (Basel). 2023 Aug 4;11(15):2207. doi: 10.3390/healthcare11152207 (PMC10418444; doi:10.3390/healthcare11152207)
Supplement: Supplementary file 1 [file healthcare-11-02207-s001.zip › healthcare-2477476-supplementary.pdf]

Effectiveness of Exercise, Cognitive Behavioral Therapy, and Pharmacotherapy on Improving Sleep in Adults with Chronic Insomnia: A Systematic Review and Network Meta-analysis of Randomized Controlled Trials

Supplementary Materials

Figure S1. PRISMA flow chart
Figure S2. Risk of bias contributions
Figure S3. Indirectness contributions

Table S1. Characteristics of interventions in the included studies
Table S2. Risk of bias for all studies
Table S3. Indirectness for all studies
Table S4. Confidence in Network Meta-analysis (CINeMA) final report
Table S5. Sensitivity analyses

S1. Search strategy
S2. Additional methodological information
S3. Pairwise meta-analyses
S4. Results on the comparative treatment effectiveness
S5. References of the included studies and details of institutional review board approval

Figure S1. PRISMA flow chart

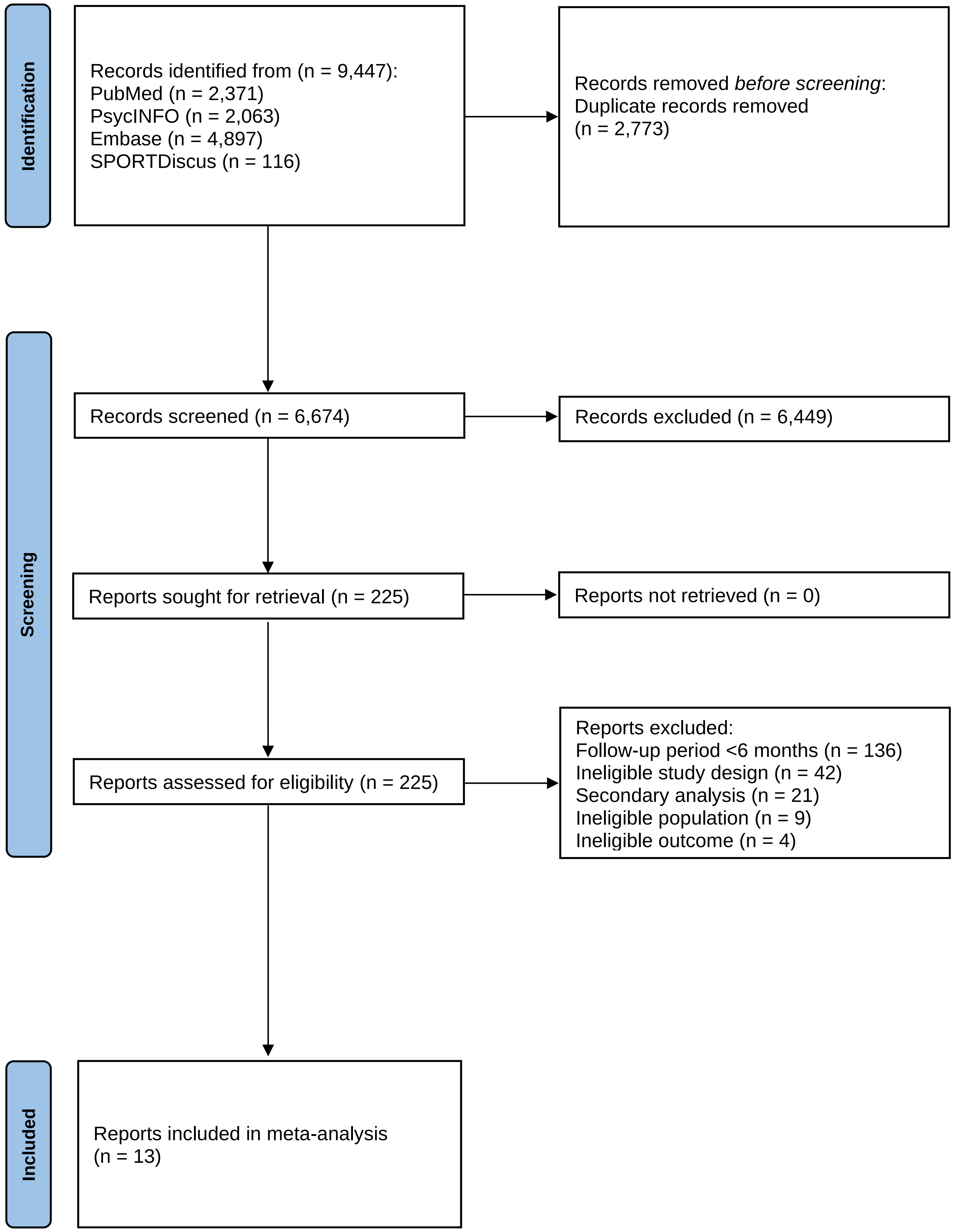

Figure S2. Risk of bias contributions

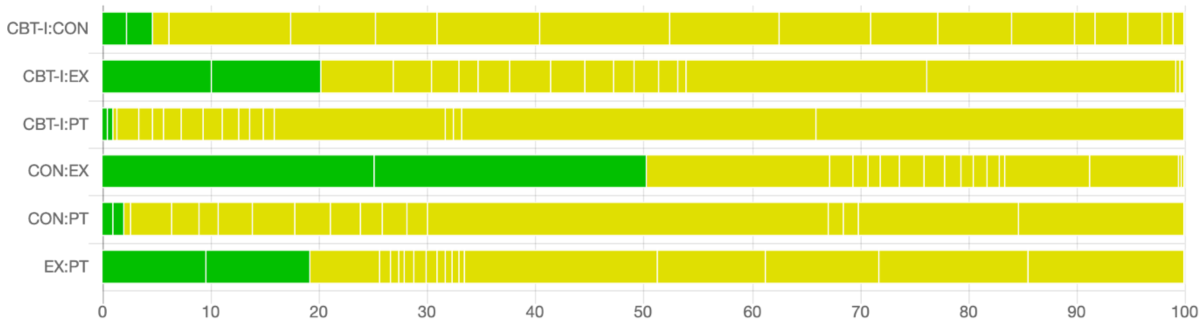

EX: exercise, CBT-I: cognitive-behavioral therapy for insomnia, PT: pharmacotherapy, CON: control.

Figure S3. Indirectness contributions

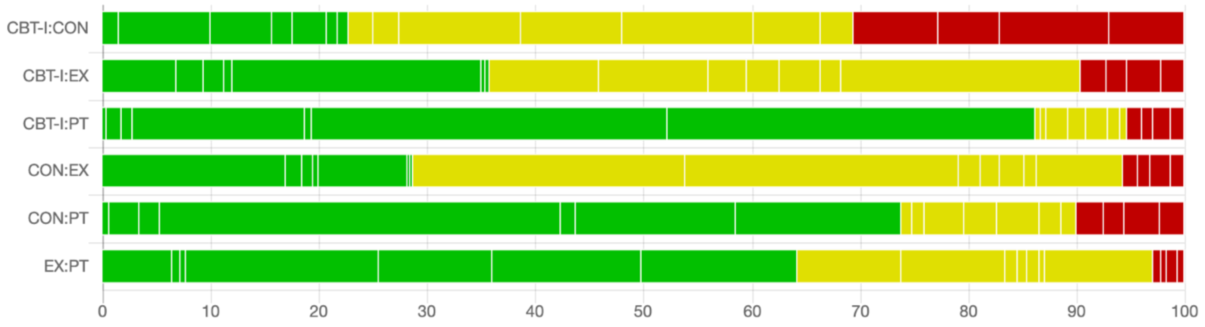

EX: exercise, CBT-I: cognitive-behavioral therapy for insomnia, PT: pharmacotherapy, CON: control.

Table S1a. Characteristics of the interventions in the included studies (two-arm)

Study	Intervention 1	Major component of intervention 1	Intervention 2	Major component of intervention 2	Intervention duration	Intervention frequency
Alessi, 2016	CBT-I	sleep restriction, stimulus control cognitive therapy, sleep hygiene relapase prevention	Sleep education*	sleep hygiene	6 weeks	5 sessions over 6 weeks
Clarke, 2015	CBT-I	sleep restriction, stimulus control cognitive therapy, psychoeducation sleep hygiene, relapse prevention	Relaxation training*	psychoeducation, muscle relaxation rapid relaxation, relapse prevention	8 weeks	Biweekly
Edinger, 2005	CBT-I	sleep restriction, stimulus control cognitive therapy	Sleep hygiene*	sleep hygiene	6 weeks	Once a week
Espie, 2007	CBT-I	sleep restriction, stimulus control cognitive therapy, relaxation sleep hygiene	Usual care*	treatment as usual	5 weeks	Once a week
Espie, 2008	CBT-I	sleep restriction, stimulus control cognitive therapy, relaxation sleep hygiene	Usual care*	treatment as usual	5 weeks	Once a week
Edinger, 2009	CBT-I	stimulus control, sleep restriction cognitive therapy, sleep hygiene	Sleep hygiene*	sleep hygiene	8 weeks	Biweekly
Järnefelt, 2020	CBT-I	sleep restriction, stimulus control cognitive therapy, relaxation sleep hygiene	Sleep hygiene*	sleep hygiene	10 weeks	6 sessions over 10 weeks

Martínez, 2014	CBT-I	sleep restriction, stimulus control<br>cognitive therapy, psychoeducation<br>relapse prevention	Sleep hygiene*	sleep hygiene	6 weeks	Once a week
Irwin,<br>2017	CBT-I	sleep restriction, stimulus control<br>cognitive therapy, relaxation<br>sleep hygiene, consolidation	Tai Chi	tai chi exercise	12 weeks	Once a week
Morin, 1999	CBT-I	sleep restriction, stimulus control<br>cognitive therapy, psychoeducation	Temazepam	temazepam pills 7.5-30 mg<br>2-3 nights, up to 7 nights per week	8 weeks	Once a week<br>(for CBT-I)

*served as the control intervention

Table S1b. Characteristics of the interventions in the included studies (three-arm)

Study	Intervention 1	Major component of intervention 1	Intervention 2	Major component of intervention 2	Intervention 3	Major component of intervention 3	Intervention duration	Intervention frequency
Irwin, 2014	CBT-I	stimulus control<br>cognitive therapy<br>psychoeducation<br>mood enhancement<br>skill consolidation	Tai chi	tai chi exercise	Sleep Seminar*	sleep hygiene	16 weeks	Once a week
Siu, 2021	Tai chi	tai chi exercise	Conventional exercise	aerobic exercise<br>muscle-strengthening exercise	Usual care*	usual care	12 weeks	Thrice a week
Wu, 2006	CBT-I	sleep restriction<br>stimulus control<br>cognitive therapy<br>sleep hygiene	Temazepam	temazepam pills<br>7.5-30 mg<br>nightly use	Placebo*	placebo pills<br>one tablet/night	8 weeks	Twice a week (for CBT-I)

*served as the control intervention

Table S2. Risk of bias of the included studies

Study	Randomization process	Deviations from the intended interventions	Missing outcome data	Measurement of the outcome	Selection of the reported results	Risk of bias
Alessi et al., 2016	Low	Low	Low	Some concerns	Low	Some concerns
Clarke et al., 2015	Low	Low	Low	Some concerns	Low	Some concerns
Edinger et al., 2005	Low	Low	Low	Some concerns	Low	Some concerns
Espie et al., 2007	Low	Low	Low	Some concerns	Low	Some concerns
Espie et al., 2008	Low	Low	Low	Some concerns	Low	Some concerns
Edinger et al., 2009	Low	Low	Low	Some concerns	Low	Some concerns
Irwin et al., 2014	Low	Low	Low	Some concerns	Low	Some concerns
Irwin et al., 2017	Low	Low	Low	Some concerns	Low	Some concerns
Järnefelt et al., 2020	Some concerns	Low	Low	Some concerns	Low	Some concerns
Morin et al., 1999	Low	Low	Low	Some concerns	Low	Some concerns
Martínez et al., 2014	Low	Low	Low	Some concerns	Low	Some concerns
Siu et al., 2021	Low	Low	Low	Low	Low	Low
Wu et al. 2006	Some concerns	Low	Low	Some concerns	Low	Some concerns

Table S3. Indirectness of the included studies

Author	Population		Intervention	Outcome		Comparisons	Indirectness
Alessi et al., 2016	Low		Low	Moderate	Self-reported questionnaire	High	Moderate
Clarke et al., 2015	High	Depression patient	Low	Moderate	Self-reported questionnaire	High	High
Edinger et al., 2005	High	Fibromyalgia patient	Low	Moderate	Self-reported questionnaire	High	High
Espie et al., 2007	Low		Low	Moderate	Self-reported questionnaire	High	Moderate
Espie et al., 2008	High	Cancer survivor	Low	Moderate	Self-reported questionnaire	High	High
Edinger et al., 2009	Low		Low	Moderate	Self-reported questionnaire	High	Moderate
Irwin et al., 2014	Low		Low	Moderate	Self-reported questionnaire	Low	Low
Irwin et al., 2017	High	Cancer survivor	Low	Moderate	Self-reported questionnaire	Low	Moderate
Järnefelt et al., 2020	Low		Low	Moderate	Self-reported questionnaire	High	Moderate
Morin et al., 1999	Low		Low	Moderate	Self-reported questionnaire	Low	Low
Martínez et al., 2014	High	Fibromyalgia patient	Low	Moderate	Self-reported questionnaire	High	High
Siu et al., 2021	Low		Low	Moderate	Self-reported questionnaire	High	Moderate
Wu et al. 2006	Low		Low	Moderate	Self-reported questionnaire	Low	Low

Table S4. Confidence in Network Meta-analysis (CINeMA) final report

Comparison	N	Within-study bias	Reporting bias	Indirectness	Imprecision	Heterogeneity	Incoherence	Confidence rating
EX vs. CON	3	Some concerns	Low risk	No concerns	No concerns	Some concerns	No concerns	High
CBT-I vs. CON	10	Some concerns	Low risk	Some concerns	No concerns	Some concerns	No concerns	High
PT vs. CON	1	Some concerns	Low risk	Some concerns	Some concerns	Some concerns	No concerns	High
EX vs. CBT-I	2	No concerns	Low risk	Some concerns	No concerns	Some concerns	No concerns	High
EX vs. PT	0	Some concerns	Low risk	Some concerns	Some concerns	No concerns	No concerns	High
CBT-I vs. PT	2	Some concerns	Low risk	No concerns	Some concerns	Some concerns	No concerns	High

N: number of pairwise comparison, EX: exercise, CBT-I: cognitive-behavioral therapy for insomnia, PT: pharmacotherapy, CON: control.

Table S5. Sensitivity analyses

Analysis	N	EX vs. CON	CBT-I vs. CON	PT vs. CON	EX vs. CBT-I	EX vs. PT	CBT-I vs. PT	I^2
Main network	18	**-.29** (-.57 to -.01)	**-.48** (-.68 to -.28)	.19 (-.32 to .69)	.19 (-.11 to .49)	-.48 (-1.03 to .08)	**-.66** (-1.15 to -.18)	55.6%
Sensitivity analysis 1	11	**-.32** (-.51 to -.13)	**-.48** (-.71 to -.24)	.40 (-.36 to 1.17)	.16 (-.08 to .40)	-.72 (-1.48 to .04)	**-.88** (-1.60 to -.16)	20.6%
Sensitivity analysis 2	14	**-.29** (-.55 to -.02)	**-.46** (-.68 to -.23)	.20 (-.29 to .69)	.17 (-.12 to .46)	-.49 (-1.02 to .05)	**-.66** (-1.13 to -.18)	52.3%
Sensitivity analysis 3	34	**-.24** (-.39 to -.09)	**-.43** (-.57 to -.30)	.21 (-.26 to .68)	.19 (-.02 to .36)	-.46 (-.94 to .03)	**-.65** (-1.11 to -.18)	52.9%

N: number of pairwise comparison, EX: exercise, CBT-I: cognitive-behavioral therapy for insomnia, PT: pharmacotherapy, CON: control.

Sensitivity 1: 11 pairwise comparisons with follow-up period ≥ 12 months were included,

Sensitivity 2: 4 pairwise comparisons with high indirectness were excluded,

Sensitivity 3: all the data generated by every scale (except one sleep diary data that was classified as an outlier and was excluded from the data analysis) in the included studies were synthesized and analyzed.

S1. Search strategy

PubMed

Search	Query
#1	Suvorexant[Title/Abstract]
#2	Eszopiclone[Title/Abstract]
#3	Zaleplon[Title/Abstract]
#4	Zolpidem[Title/Abstract]
#5	Triazolam[Title/Abstract]
#6	Temazepam [Title/Abstract]
#7	Ramelteon[Title/Abstract]
#8	Doxepin[Title/Abstract]
#9	Hypnotic*[Title/Abstract] OR Soporific*[Title/Abstract] OR "Sleep* medication*"[Title/Abstract]
#10	Exercise[MeSH Terms] OR exercise[Title/Abstract] OR "physical activity"[Title/Abstract] OR aerobic[Title/Abstract] OR running[Title/Abstract] OR walk*[Title/Abstract] OR jogging[Title/Abstract] OR swim*[Title/Abstract] OR cycl*[Title/Abstract] OR taichi [Title/Abstract] OR tai chi [Title/Abstract] OR yoga [Title/Abstract] OR qigong [Title/Abstract] OR qi gong*[Title/Abstract] OR "mind-body" [Title/Abstract] OR "mind body" [Title/Abstract]
#11	Psychotherapy[Title/Abstract] OR "behavior* therapy" [Title/Abstract] OR cognitive therapy" OR "behaviour* therapy" [Title/Abstract]
#12	#1 OR #2 OR #3 OR #4 OR #5 OR #6 OR #7 OR #8 OR #9
#13	Insomnia[MeSH Terms] OR insomnia*[Title/Abstract] OR "Sleep* disturbance*" [Title/Abstract] OR "Sleep* disorder*" [Title/Abstract] OR Sleep* [Title/Abstract]
#14	Randomized controlled trial[MeSH Terms] OR Random allocation[MeSH Terms] OR (random*[Title/Abstract] AND (control*[Title/Abstract] OR placebo[Title/Abstract]))
#15	Adults[MeSH Terms] OR adult*[Title/Abstract]
#16	#12 OR #10 OR #11
#17	#16 AND #13 AND #14 AND #15
#18	Filters: from 1990 - 3000/12/12

PsycINFO

Search	Query
#1	SU(suvorexant) OR AB(suvorexant) OR TI(suvorexant)
#2	SU(eszopiclone) OR AB(eszopiclone) OR TI(eszopiclone)
#3	SU(zaleplon) OR AB(zaleplon) OR TI(zaleplon)
#4	SU(zolpidem) OR AB(zolpidem) OR TI(zolpidem)
#5	SU(triazolam) OR AB(triazolam) OR TI(triazolam)
#6	SU(temazepam) OR AB(temazepam) OR TI(temazepam)
#7	SU(ramelteon) OR AB(ramelteon) OR TI(ramelteon)
#8	SU(doxepin) OR AB(doxepin) OR TI(doxepin)
#9	SU(hypnotic*) OR AB(hypnotic*) OR TI(hypnotic*)
#10	SU(soporific*) OR AB(soporific*) OR TI(soporific*)
#11	SU(sleep* medication*) OR AB(sleep* medication*) OR TI(sleep* medication*)
#12	SU(exercise) OR TI(exercise) OR AB(exercise) OR SU(physical activity) OR TI(physical activity) OR AB(physical activity) OR TI(aerobic) OR AB(aerobic) OR TI(running) OR AB(running) OR TI(walk*) OR AB(walk*) OR TI(jogging) OR AB(jogging) OR TI(swim*) OR AB(swim*) OR TI(cycl*) OR AB(cycl*) OR TI(taichi) OR AB(taichi) OR TI(yoga) OR AB (yoga) OR TI(qigong) OR AB (qigong) OR TI(qi gong) OR AB (qi gong) OR TI(mind-body) OR AB (mind-body) OR TI(mind body) OR AB (mind body)
#13	TI(psychotherapy*) OR AB (psychotherapy*) OR TI(behavior* therapy) OR AB (behavior* therapy) OR TI(behaviour* therapy) OR AB (behaviour* therapy) OR TI(cognitive therapy) OR AB (cognitive therapy)
#14	S1 OR S2 OR S3 OR S4 OR S5 OR S6 OR S7 OR S8 OR S9 OR S10 OR S11
#15	S12 OR S13 OR S14
#16	SU(insomnia) OR SU(chronic insomnia) OR TI(insomnia*) OR AB(insomnia*) OR TI(sleep* disturbance*) OR AB(sleep* disturbance*) OR TI(sleep* disorder*) OR AB(sleep* disorder*) OR TI(sleep*) OR AB(sleep*)
#17	SU(randomized controlled trial) OR SU(randomized clinical trial) OR SU(random allocation) OR (random* AND control*) OR (random* AND placebo)
#18	S15 AND S16 AND S17
#19	SU(adult*) OR TI(adult*) OR AB(adult*)
#20	S18 AND S19
#21	#20 Limit date range 1990-2022

Embase

Search	Query
#1	exp suvorexant/ or suvorexant.tn,ab,ti.
#2	exp eszopiclone/ or eszopiclone.tn,ab,ti.
#3	exp zaleplon/ or zaleplon.tn,ab,ti.
#4	exp zolpidem/ or zolpidem.tn,ab,ti.
#5	exp triazolam/ or triazolam.tn,ab,ti.
#6	exp temazepam/ or temazepam.tn,ab,ti.
#7	exp ramelteon/ or ramelteon.tn,ab,ti.
#8	exp doxepin/ or doxepin.tn,ab,ti.
#9	exp hypnotic agent/ or hypnotic*.ti,ab.
#10	exp soporific/ or soporific*.ti,ab.
#11	1 or 2 or 3 or 4 or 5 or 6 or 7 or 8 or 9 or 10
#12	exp exercise/ or exercise.ti,ab. or exp physical activity/ or physical activity.ti,ab. or aerobic.ti,ab. or running.ti,ab. or walk*.ti,ab. or jogging.ti,ab. or swim*.ti,ab. or cycl*.ti,ab. or taichi.ti,ab. or tai chi.ti,ab. or yoga.ti,ab. or qigong.ti,ab. or qi gong.ti,ab. or mind-body.ti,ab. or mind body.ti,ab.
#13	Psychotherapy*.ti,ab. or behavior* therapy.ti,ab. or behaviour* therapy.ti,ab. or cognitive therapy.ti,ab.
#14	11 or 12 or 13
#15	exp insomnia/ or exp sleep disturbance/ or exp sleep disorder/ or exp sleep
#16	exp randomized controlled trial/ or randomized controlled trial.ab,ti,pt. or randomized placebo trial.ti,ab. or exp randomization/
#17	exp adult/ or adult*.ti,ab.
#18	14 and 15 and 16 and 17
#19	Limit 19 to yr=1990-Current

EBSCOhost SPORTDiscus

Search	Query
#1	TI (suvorexant) OR AB (suvorexant)
#2	TI (eszopiclone) OR AB (eszopiclone)
#3	TI (zaleplon) OR AB (zaleplon)
#4	TI (zolpidem) OR AB (zolpidem)
#5	TI (triazolam) OR AB (triazolam)
#6	TI (temazepam) OR AB (temazepam)
#7	TI (ramelteon) OR AB (ramelteon)
#8	TI (doxepin) OR AB (doxepin)
#9	TI (hypnotic agent) OR AB (hypnotic agent)
#10	TI (soporific*) OR AB (soporific*)
#11	S1 or S2 or S3 or S4 or S5 or S6 or S7 or S8 or S9 or S10
#12	DE “EXERCISE”
#13	DE “EXERCISE psychology”
#14	DE “EXERCISE physiology”
#15	DE “EXERCISE therapy”
#16	DE “PHYSICAL activity”
#17	DE “PHYSICAL fitness”
#18	TI (physical-activit* OR exercis* OR sport* OR fitness OR aerobic* OR walk* OR jogging OR swim* OR cycl* OR yoga OR tai chi OR taichi OR qigong OR qi gong OR mind-body OR mind body) OR AB (physical-activit* OR exercis* OR sport* OR fitness OR aerobic* OR walk* OR jogging OR swim* OR cycl* OR yoga OR tai chi OR taichi OR qigong OR qi gong OR mind-body OR mind body) OR KW (physical-activit* OR exercis* OR sport* OR fitness OR aerobic* OR walk* OR jogging OR swim* OR cycl* OR yoga OR tai chi OR taichi OR qigong OR qi gong OR mind-body OR mind body) OR SU (physical-activit* OR exercis* OR sport* OR fitness OR aerobic* OR walk* OR jogging OR swim* OR cycl* OR yoga OR tai chi OR taichi OR qigong OR qi gong OR mind-body OR mind body)
#19	S12 OR S13 OR S14 OR S15 OR S16 OR S17 OR S18 OR S19
#20	DE (PSYCHOTHERAPY)
#21	DE (BEHAVIOR therapy)
#22	DE (BEHAVIOUR therapy)
#23	DE (COGNITIVE therapy)
#24	S21 OR S22 OR S23 OR S24
#25	DE “SLEEP disorders”
#26	TI (insomnia* OR sleep disturbance OR sleep disorder or sleep) OR AB (insomnia* OR sleep disturbance OR sleep disorder or sleep) OR KW (insomnia* OR sleep disturbance OR sleep disorder or sleep) OR SU (insomnia* OR sleep disturbance OR sleep disorder or sleep)
#27	TI (randomi#ed)
#28	AB (random* OR controlled)
#29	TI (trial)
#30	AB (assigned OR allocated)
#31	DE “PLACEBOS”
#32	TI (placebo) OR AB (placebo)
#33	S28 OR S29 OR S30 OR S31 OR S32 OR S33
#34	S11 OR S20 OR 25
#35	S26 OR S27
#36	S34 AND S35

S2. Additional methodological information

2.1 Risk of bias

The quality of the included studies was evaluated by the Cochrane risk of bias assessment tool (RoB-2) [74] across five domains: 1) randomization process, 2) deviations from intended interventions, 3) missing outcome data, 4) measurement of the outcome, and 5) selection of the reported results. Each domain was rated as having low risk of bias, some concerns, or high risk of bias. To implement RoB-2, we used the Excel template provided in the Cochrane Handbook for Systematic Reviews of Interventions [74], which provides signalling questions for risk of bias assessment.

1) The randomization process was assessed using three signalling questions: 1.1) was the allocation sequence random? 1.2) was the allocation sequence concealed until participants were enrolled and assigned to interventions? 1.3) did baseline differences between intervention groups suggest a problem with the randomization process?

2)The deviations from intended interventions were assessed using seven signalling questions: 2.1) were participants aware of their assigned intervention during the trial? 2.2) were carers and people delivering the interventions aware of participants' assigned intervention during the trial? 2.3) were there deviations from the intended intervention that arose because of the trial context? 2.4) were these deviations likely to have affected the outcome? 2.5) were these deviations from intended intervention balanced between groups? 2.6) was an appropriate analysis used to estimate the effect of assignment to intervention? 2.7) was there potential for a substantial impact (on the result) of the failure to analyse participants in the group to which they were randomized?

3) The missing outcome data was assessed using four signalling questions: 3.1) were data for this outcome available for all, or nearly all, participants randomized? 3.2) is there evidence that the result was not biased by missing outcome data? 3.3) could missingness in the outcome depend on its true value? 3.4) is it likely that missingness in the outcome depended on its true value?

4) The measurement of the outcome was assessed using five signalling questions: 4.1) was the method of measuring the outcome inappropriate? 4.2) could measurement or ascertainment of the outcome have differed between intervention groups? 4.3) were outcome assessors aware of the intervention received by study participants? 4.4) could assessment of the outcome have been influenced by knowledge of intervention received? 4.5) Is it likely that assessment of the outcome was influenced by knowledge of intervention received?

5) The selection of the reported results was assessed using three signalling questions: 5.1) were the data that produced this result analysed in accordance with a pre-specified analysis plan that was finalized before unblinded outcome data were available for analysis? 5.2) Is the numerical result being assessed likely to have been selected, on the basis of the results, from multiple eligible outcome measurements (e.g., scales, definitions, time points) within the outcome domain? 5.3) Is the numerical result being assessed likely to have been selected, on the basis of the results, from multiple eligible analyses of the data?

The assessments were conducted by two independent researchers. A third researcher was consulted to mediate any disagreements, which were resolved by consensus. Each domain was evaluated by the algorithm embedded in the Excel provided by the RoB-2.

2.2 Indirectness

Indirectness was assessed based on the Grading of Recommendations, Assessment, Development and Evaluations (GRADE) framework [75-76]. The included studies were assessed on four outcomes: 1) population, 2) intervention, 3) outcome, and 4) whether a study showed direct evidence for at least one comparison of interest.

Study indirectness was rated as "low" if ≥ 3 outcomes were considered to be low, "unclear if ≤ 1 outcome was unclear, and "high" if ≥ 2 outcomes were considered to be high. Any other combination was rated as "moderate".

2.3 Confidence of Network Meta-analysis (CINeMA) rating

We used the CINeMA framework to assess the confidence in the cumulative evidence [77]. The CINeMA web application of the Grading of Recommendations, Assessment, Development, and Evaluations (GRADE) covers six domains: 1) within-study bias, 2) reporting bias, 3) indirectness, 4) imprecision, 5) heterogeneity, and 6) incoherence.

1) Within-study bias was evaluated using the RoB-2 methods described in section 2.1.

2) Reporting bias was rated as "suspected" or "undetected" based on the entirety of the research and the available data.

3) Indirectness was assessed using the methods described in section 2.2.

4 to 6) Imprecision, heterogeneity, and incoherence, were assessed with the clinically significant effect size set to -0.4. The selection was based on a meta-analysis by Zweerde and colleagues [78]. Their study investigated the long-term effectiveness of CBT-I on improving sleep, which reported an effect size of -0.4 for CBT-I at ≥6 months post-intervention. There are few studies on the long-term effectiveness of pharmacotherapy and exercise on improving sleep. We believe our selection of the clinically significant effect size of -0.4 will provide a stringent evaluation on the imprecision, heterogeneity, and incoherence assessment with respect to CBT-I, as it is considered to be the first-line treatment for long-term insomnia management [79].

References for section 2.1 to 2.3:

74. Sterne JA, Savović J, Page MJ, Elbers RG, Blencowe NS, Boutron I, Cates CJ, Cheng HY, Corbett MS, Eldridge SM, Emberson JR. RoB 2: a revised tool for assessing risk of bias in randomised trials. Bmj. 2019 Aug 28;366.

75. Guyatt GH, Oxman AD, Vist GE, Kunz R, Falck-Ytter Y, Alonso-Coello P, Schünemann HJ. GRADE: an emerging consensus on rating quality of evidence and strength of recommendations. Bmj. 2008 Apr 24;336(7650):924-6.

76. Guyatt GH, Oxman AD, Kunz R, Woodcock J, Brozek J, Helfand M, Alonso-Coello P, Falck-Ytter Y, Jaeschke R, Vist G, Akl EA. GRADE guidelines: 8. Rating the quality of evidence - indirectness. Journal of clinical epidemiology. 2011 Dec 1;64(12):1303-10.

77. Nikolakopoulou A, Higgins JP, Papakonstantinou T, Chaimani A, Del Giovane C, Egger M, Salanti G. CINeMA: an approach for assessing confidence in the results of a network meta-analysis. PLoS medicine. 2020 Apr 3;17(4):e1003082.

78. Van der Zweerde T, Bisdounis L, Kyle SD, Lancee J, van Straten A. Cognitive behavioral therapy for insomnia: a meta-analysis of long-term effects in controlled studies. Sleep Medicine Reviews. 2019 Dec 1;48:101208.

79. Qaseem A, Kansagara D, Forciea MA, Cooke M, Denberg TD, Clinical Guidelines Committee of the American College of Physicians*. Management of chronic insomnia disorder in adults: a clinical practice guideline from the American College of Physicians. Annals of internal medicine. 2016 Jul 19;165(2):125-33.

S3. Pairwise meta-analyses

1. Comparative long-term effectiveness of the interventions on sleep

Comparison a: Exercise vs. Control

Study	Experimental Total	Experimental Mean	Experimental SD	Control Total	Control Mean	Control SD	SMD	95%-CI	Weight
Siu 2021(2)	105	8.90	5.6000	110	11.30	5.2000	-0.44	[-0.71; -0.17]	43.2%
Siu 2021	105	9.10	5.3000	110	11.30	5.2000	-0.42	[-0.69; -0.15]	43.3%
Irwin 2014	48	7.00	4.7000	25	7.60	4.4000	-0.13	[-0.61; 0.35]	13.5%
**Random effects model**	**258**			**245**			**-0.39**	**[-0.70; -0.07]**	**100.0%**

Heterogeneity: $I^2 = 0\%$, $\tau^2 = 0$, $p = 0.52$

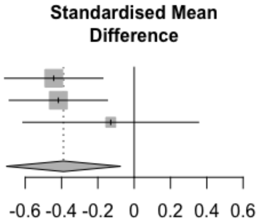

Comparison b: CBT-I vs. Control

Study	Experimental Total	Experimental Mean	Experimental SD	Control Total	Control Mean	Control SD	SMD	95%-CI	Weight
Clarke 2015	32	7.90	5.7000	32	14.40	4.8000	-1.22	[-1.75; -0.68]	9.5%
Wu 2006	19	-83.90	12.7000	17	-72.40	13.2000	-0.87	[-1.56; -0.18]	7.7%
Espie 2007	107	8.40	4.1400	94	11.20	3.2400	-0.74	[-1.03; -0.46]	13.1%
Irwin 2014	50	5.40	3.1000	25	7.60	4.4000	-0.61	[-1.10; -0.12]	10.2%
Edinger 2005	16	-89.00	4.4000	17	-85.30	7.8300	-0.56	[-1.26; 0.13]	7.6%
Martinez 2014	27	11.63	4.6300	20	13.30	4.1500	-0.37	[-0.95; 0.21]	8.9%
Alessi 2016	106	6.50	5.5600	53	7.50	3.7100	-0.20	[-0.53; 0.13]	12.5%
Espie 2008	68	-89.80	8.8100	39	-88.50	13.9300	-0.12	[-0.51; 0.28]	11.6%
Edinger 2009	41	8.00	5.1200	40	7.60	5.0600	0.08	[-0.36; 0.51]	10.9%
Jarnefelt 2020	20	-85.71	18.2700	17	-89.38	15.6000	0.21	[-0.44; 0.86]	8.1%
**Random effects model**	**486**			**354**			**-0.43**	**[-0.74; -0.12]**	**100.0%**

Heterogeneity: $I^2 = 68\%$, $\tau^2 = 0.1224$, $p < 0.01$

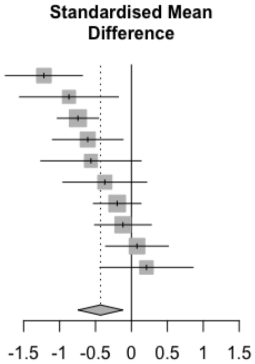

Comparison c: Pharmacotherapy vs. Control

Study	Experimental Total	Experimental Mean	Experimental SD	Control Total	Control Mean	Control SD	SMD	95%-CI
Wu 2006	17	-74.40	9.5000	17	-72.40	13.2000	-0.17	[-0.84; 0.5]

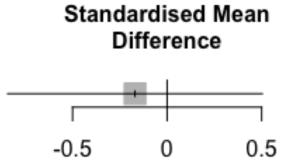

Comparison d: Exercise vs. CBT-I

Study	Experimental Total	Experimental Mean	Experimental SD	Control Total	Control Mean	Control SD	SMD	95%-CI	Weight
Irwin 2017	45	7.40	3.3500	45	6.80	2.6800	0.20	[-0.22; 0.61]	48.9%
Irwin 2014	48	8.00	3.4000	50	6.40	2.6000	0.53	[0.12; 0.93]	51.1%
**Random effects model**	**93**			**95**			**0.36**	**[-1.73; 2.46]**	**100.0%**

Heterogeneity: $I^2 = 20\%$, $\tau^2 = 0.0109$, $p = 0.26$

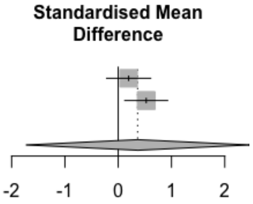

Comparison e: CBT-I vs. Pharmacotherapy

Study	Experimental Total	Experimental Mean	Experimental SD	Control Total	Control Mean	Control SD	SMD	95%-CI	Weight
Morin 1999	18	-84.70	12.2000	17	-75.28	8.2000	-0.88	[-1.58; -0.18]	49.0%
Wu 2006	19	-83.90	12.7000	17	-74.40	9.5000	-0.82	[-1.51; -0.14]	51.0%
**Random effects model**	**37**			**34**			**-0.85**	**[-1.23; -0.48]**	**100.0%**

Heterogeneity: $I^2 = 0\%$, $\tau^2 = 0$, $p = 0.91$

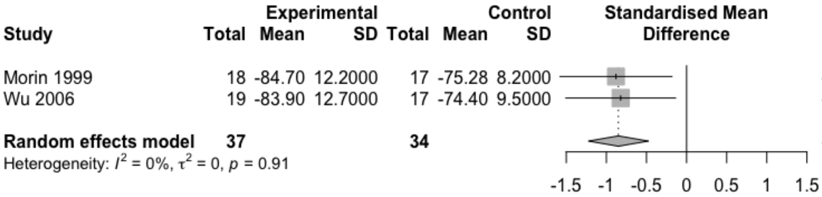

2. Comparative post-intervention treatment effectiveness of the interventions on sleep

Comparison a: Exercise vs. Control

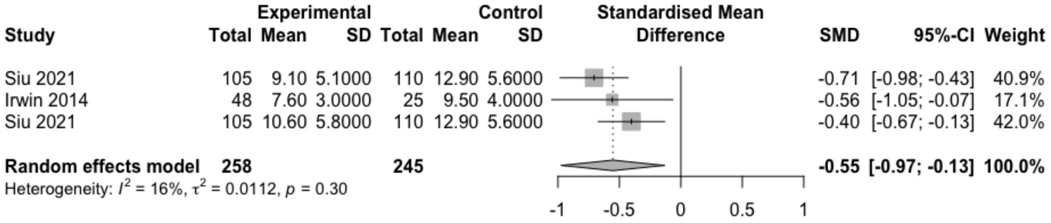

Comparison b: CBT-I vs. Control

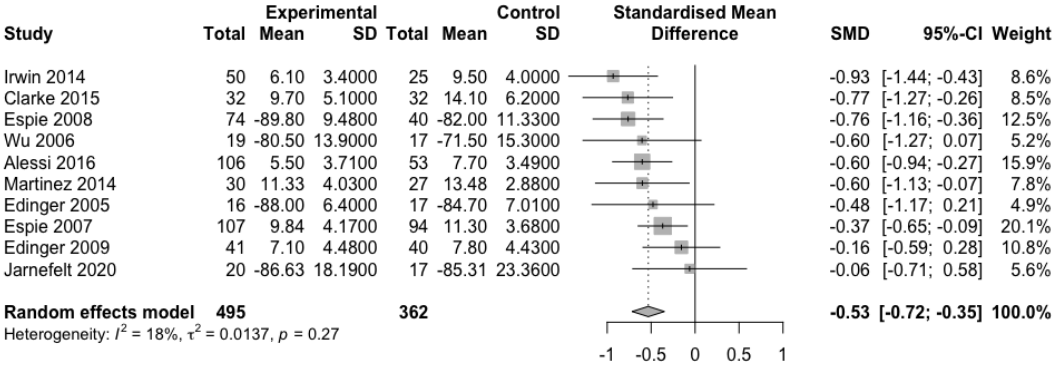

Comparison c: Pharmacotherapy vs. Control

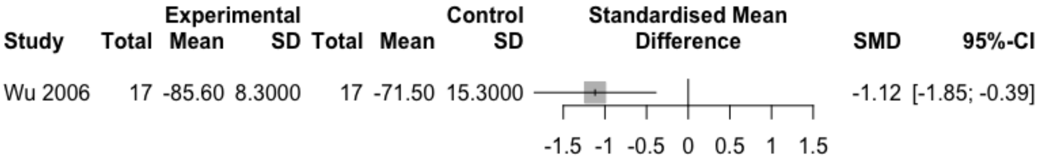

Comparison d: Exercise VS. CBT-I

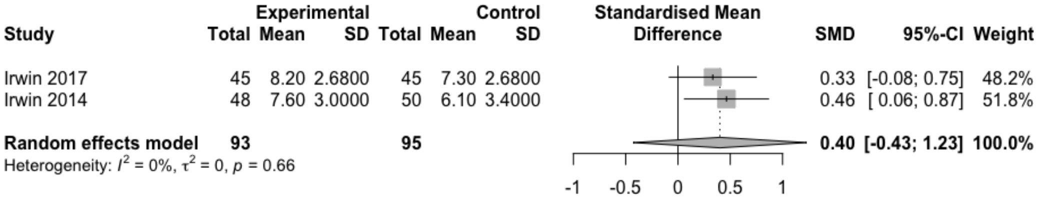

Comparison e: CBT-I vs. Pharmacotherapy

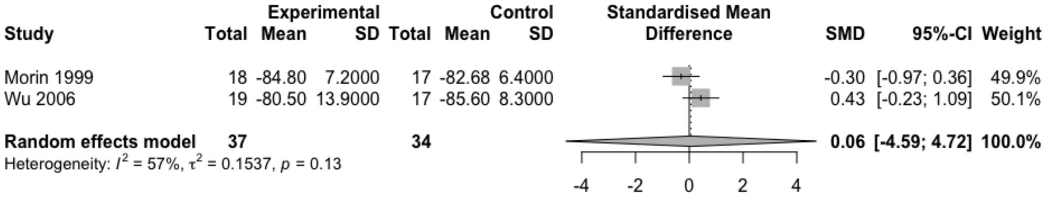

S4. Results of the comparative post-intervention treatment effectiveness of the interventions from the network and pairwise meta-analyses

**Exercise**	0.40 (-0.43 to 1.23) (N=2; I^2=0.0%)	NA[b]	**-0.55** (-0.97 to -0.13) (N=2; I^2=16.1%)
0.16 (-0.07 to 0.40) (N=13; I^2=32.4%)	**CBT-I**	0.06 (-4.59 to 4.72) (N=2, I^2=57.2%)	**-0.53** (-0.72 to -0.35) (N=10, I^2=18.3%)
0.37 (-0.85 to 0.12) (N=13; I^2=32.4%)	-0.20 (-0.30 to 0.70) (N=13; I^2=32.4%)	**Temazepam**	**-1.12** (-1.85 to -0.39) (N=1; I^2=NA[a])
**-0.44** (-0.65 to -0.22) (N=13; I^2=32.4%)	**-0.60** (-0.76 to -0.44) (N=13; I^2=32.4%)	**-0.80** (-1.25 to -0.36) (N=13; I^2=32.4%)	**Control**

Results of the network meta-analyses are presented in grey boxes, and results of the pairwise meta-analyses are presented in white boxes. Estimates are displayed as column vs. row for the network meta-analyses and row vs. column for the pairwise meta-analyses. Results are expressed as SMDs. A negative SMD indicates superiority of the first treatment over the comparison treatment. Bold estimates indicate a statistical difference.

N=number of studies in the comparison

NA[a]=no evidence on I^2 was available as there was only one study for that comparison.

NA[b]=no studies compared exercise vs. temazepam.

S5. **References of the included studies and details of institutional review board approval**

1. Alessi, C., Martin, J.L., Fiorentino, L., Fung, C.H., Dzierzewski, J.M., Rodriguez Tapia, J.C., Song, Y., Josephson, K., Jouldjian, S. and Mitchell, M.N., 2016. Cognitive behavioral therapy for insomnia in older veterans using nonclinician sleep coaches: randomized controlled trial. Journal of the American Geriatrics Society, 64(9), pp.1830-1838.

   IRB Approval: The Veterans Affairs (VA) Greater Los Angeles Healthcare System.

2. Norell-Clarke, A., Jansson-Fröjmark, M., Tillfors, M., Holländare, F. and Engström, I., 2015. Group cognitive behavioural therapy for insomnia: Effects on sleep and depressive symptomatology in a sample with comorbidity. Behaviour research and therapy, 74, pp.80-93.

   IRB Approval: The Regional Ethics Vetting Board in Uppsala, Sweden.

3. Edinger, J.D., Wohlgemuth, W.K., Krystal, A.D. and Rice, J.R., 2005. Behavioral insomnia therapy for fibromyalgia patients: a randomized clinical trial. Archives of internal medicine, 165(21), pp.2527-2535.

   IRB Approval: Duke University Medical Centre’s Institutional Review Board.

4. Espie, C.A., MacMahon, K.M., Kelly, H.L., Broomfield, N.M., Douglas, N.J., Engleman, H.M., McKinstry, B., Morin, C.M., Walker, A. and Wilson, P., 2007. Randomized clinical effectiveness trial of nurse-administered small-group cognitive behavior therapy for persistent insomnia in general practice. Sleep, 30(5), pp.574-584.

   IRB Approval: Local NHS Research Ethics Committees.

5. Espie, C.A., Fleming, L., Cassidy, J., Samuel, L., Taylor, L.M., White, C.A., Douglas, N.J., Engleman, H.M., Kelly, H.L. and Paul, J., 2008. Randomized controlled clinical effectiveness trial of cognitive behavior therapy compared with treatment as usual for persistent insomnia in patients with cancer. Journal of clinical oncology, 26(28), pp.4651-4658.

   IRB Approval: Local NHS Research Ethics Committees.

6. Edinger, J.D., Olsen, M.K., Stechuchak, K.M., Means, M.K., Lineberger, M.D., Kirby, A., and Carney, C.E., 2009. Cognitive behavioral therapy for patients with primary insomnia or insomnia associated predominantly with mixed psychiatric disorders: a randomized clinical trial. Sleep, 32(4), pp.499-510.

   IRB Approval: The Institutional Review Board of the Durham (NC) VA Medical Center.

7. Irwin, M.R., Olmstead, R., Carrillo, C., Sadeghi, N., Breen, E.C., Witarama, T., Yokomizo, M., Lavretsky, H., Carroll, J.E., Motivala, S.J. and Bootzin, R., 2014. Cognitive behavioral therapy vs. Tai Chi for late life insomnia and inflammatory risk: a randomized controlled comparative efficacy trial. Sleep, 37(9), pp.1543-1552.

   IRB Approval: UCLA Institutional Review Board.

8. Irwin, M.R., Olmstead, R., Carrillo, C., Sadeghi, N., Nicassio, P., Ganz, P.A. and Bower, J.E., 2017. Tai Chi Chih compared with cognitive behavioral therapy for the treatment of insomnia in survivors of breast cancer: a randomized, partially blinded, noninferiority trial. Journal of Clinical Oncology, 35(23), p.2656.

   IRB Approval: UCLA Institutional Review Board.

9. Järnefelt, H., Härmä, M., Sallinen, M., Virkkala, J., Paajanen, T., Martimo, K.P. and Hublin, C., 2020. Cognitive behavioural therapy interventions for insomnia among shift workers: RCT in an occupational health setting. International archives of occupational and environmental health, 93(5), pp.535-550.

IRB Approval: The Coordinating Ethics Committees of the Hospital District of Helsinki and Uusimaa, Finland.

10. Morin, C.M., Colecchi, C., Stone, J., Sood, R. and Brink, D., 1999. Behavioral and pharmacological therapies for late-life insomnia: a randomized controlled trial. Jama, 281(11), pp.991-999.

IRB Approval: The Institutional Review Board of the Medical College of Virginia/ Virginia Commonwealth University.

11. Martínez, M.P., Miró, E., Sánchez, A.I., Díaz-Piedra, C., Cáliz, R., Vlaeyen, J.W. and Buela-Casal, G., 2014. Cognitive-behavioral therapy for insomnia and sleep hygiene in fibromyalgia: a randomized controlled trial. Journal of behavioral medicine, 37(4), pp.683-697.

IRB Approval: The University of Granada Ethics Committee.

12. Siu, P.M., Angus, P.Y., Tam, B.T., Chin, E.C., Doris, S.Y., Chung, K.F., Hui, S.S., Woo, J., Fong, D.Y., Lee, P.H. and Wei, G.X., 2021. Effects of tai chi or exercise on sleep in older adults with insomnia: a randomized clinical trial. JAMA network open, 4(2), pp.e2037199-e2037199.6 s

IRB Approval: The Hong Kong Polytechnic University Human Subjects Ethics Office.

13. Wu, R., Bao, J., Zhang, C., Deng, J. and Long, C., 2006. Comparison of sleep condition and sleep-related psychological activity after cognitive-behavior and pharmacological therapy for chronic insomnia. Psychotherapy and psychosomatics, 75(4), pp.220-228.

IRB Approval: The Institutional Review Board of Peking University.
